# Neuroprotective effects of crocin and crocin-loaded niosomes against the paraquat-induced oxidative brain damage in rats

**DOI:** 10.1515/biol-2022-0468

**Published:** 2022-09-14

**Authors:** Afsoon Daneshvar, Ali Fathi Jouzdani, Farzin Firozian, Sara Soleimani Asl, Mojdeh Mohammadi, Akram Ranjbar

**Affiliations:** Department of Pharmacology and Toxicology, School of Pharmacy, Hamadan University of Medical Sciences, Hamadan, 6517838678, Iran; Department of Neuroscience, Neuroscience and Artificial Intelligence Research Group (NAIRG), Student Research Committee, Hamadan University of Medical Sciences, Hamadan, Iran; USERN Office, Hamadan University of Medical Sciences, Hamadan, Iran; Department of Pharmaceutics, Faculty of Pharmacy, Hamadan University of Medical Science, Hamadan, Iran; Department of Anatomy, School of Medicine, Hamadan University of Medical Sciences, Hamadan, Iran

**Keywords:** brain, crocin-loaded niosomes, oxidative stress, paraquat, rats

## Abstract

Paraquat (PQ) is a nonselective herbicide that induces oxidative reactions and multiple-organ failure on exposure. Crocin, a carotenoid obtained from saffron, has demonstrated many therapeutic effects against neural conditions because of its antioxidant properties. In this study, 30 male Wistar rats were divided into 6 groups to evaluate the protective effects of crocin and crocin-loaded niosomes (NC) against PQ in the brain. The levels of total antioxidant capacity (TAC), lipid peroxidation (LPO), total thiol groups (TTG), superoxide dismutase (SOD), and catalase (CAT) activity were measured as the markers of redox status. Histopathological changes in the CA1 region of the hippocampus were evaluated by cresyl violet staining. Results indicated that both crocin and NC were able to attenuate the adverse effects of PQ at the histopathological level, which was following the changes in LPO (*P* < 0.0001), TAC (*P* < 0.01), and TTG (*P* < 0.05) level. The activity of CAT (*P* < 0.01) and SOD (*P* < 0.01) could be restored either by crocin or NC. Also, results indicated that nanoformulation of crocin in niosomes appears to be more promising. In conclusion, both crocin and NC showed favourable effects of PQ in the brain of rats, and were determined to be excellent agents to prevent acute toxicities of PQ. Furthermore, these two compounds can be known to provide neuroprotection.

## 1 Introduction

Paraquat (PQ) (*N*,*N*′-dimethyl-4,4′-bipyridinium dichloride) is a nonselective herbicide that acts quickly and spreads to organs instantaneously, leading to oxidative reactions and multiple-organ failure [1]. Having no specific chelator, antidote, or medication to attenuate toxic effects has elevated PQ exposure mortality rates [2]. Although the toxicity mechanism is not precise, it is concluded that PQ exerts its neurotoxicity via the formation of reactive oxygen species, oxidizing the reducing components such as NADPH and reduced glutathione, and finally leading to cellular damage [3,4]. Accordingly, antioxidant agents have been greatly emphasised as treatment modalities for PQ exposure [5]. It has been shown that PQ is highly associated with neurodegenerative diseases, especially Parkinson’s disease [6]. Hence, this herbicide is used to develop Parkinson’s disease animal models [7]. The importance of this is the ability to modulate oxidative stress caused by herbicides could be used for neurodegenerative disease as a helpful treatment. Saffron, a food colourant obtained from the stigma of a perennial flower named *Crocus sativus*, is introduced to have beneficial effects [8]. Crocin (mono-glycosyl or di-glycosyl polyene esters), constituting 6–16% of saffron dry matter, is responsible for saffron colour [9]. Research interests have been attracted to principal phytochemicals derived from saffron, including crocin (or crocin-1), which demonstrates numerous therapeutic properties against neurodegenerative diseases, diabetes, and inflammation [10]. The previous studies suggested that crocin bears protective effects against many natural and chemical toxicities, including lipopolysaccharides [11], aflatoxins [12], cyclophosphamide [13], and metals [14]. Furthermore, evidence state that crocin could protect the brain in oxidative states caused by carbon tetrachloride [15], diabetes [16], or ethidium bromide [17]. Crocin is soluble in water yet sensitive to thermal degradation. Therefore, it could benefit from novel strategies. Also, literature shows that crocin is scarcely absorbed after gavage [18]. Nanomaterials with the size of typically less than 100 nm bearing excellent surface-area-to-volume ratios have enhanced the efficiency of chemical agents, including saffron components [19]. This work studied the antioxidant effects of crocin and crocin-loaded niosomes (NC) in brain tissues of male rats treated with PQ.

## 2 Material and methods

### 2.1 Niosome

As described previously, NC was prepared by the “Surface active agents film hydration” method. Span-60, polyethene glycol, and cholesterol were dissolved in ethanol. A rotary eliminated ethanol under vacuum, and then phosphate buffer was added and stirred to apply hydration. The mixture was sonicated in the buffer.

### 2.2 *In vivo* study

#### 2.2.1 Animal treatment

Thirty male Wistar rats (180–250 g) were acquired from the animal colony of the Hamadan University of Medical Sciences, Hamadan, Iran. Animals were put under approved environmental circumstances of 22 ± 1°C temperature, 45–55% humidity, and 12/12 h light/dark cycle, with sufficient water and food supply. According to previous study by Samarghandian et al. [20], the appropriate dose of Crocin was determined. Animals were randomly divided into six groups of five as follows: the control group was injected with normal saline, the second group was treated with crocin at 20 mg/kg/day for 7 days by intraperitoneal injection (IP), and the third group was treated with PQ at a dose of 5 mg/kg/day for 7 days (IP), the fourth group was treated with PQ at a quantity of 5 mg/kg/day and with crocin at an amount of 20 mg/kg/day for 7 days, the fifth group was treated with NC at a dose of 20 mg/kg/day for 7 days, and the sixth group was treated with PQ at a quantity of 5 mg/kg/day and treated with NC at a dose of 20 mg/kg/day for 7 days.

Twenty-four hours following the final treatment, rats were anaesthetised with an IP injection of ketamine/xylazine. Brain tissues were separated and cleansed with a saline solution immediately. One hemisphere was maintained in 10% formalin for histopathological studies, and the other was frozen in liquid nitrogen and stored at −70°C for biochemical analysis. Brain allocations were homogenised in 1:1 volumes of PBS (pH 7.4). The sequent homogenate was centrifuged at 10,000*g* for 15 min. After that, the supernatant was stored at –70°C as brain homogenate for additional biochemical assays.


**Ethical approval:** The research related to animal use has been complied with all the relevant national regulations and institutional policies for the care and use of animals, and was approved by The Ethics Committee Guidelines of Hamadan University of Medical Sciences (Res: IR.UMSHA.REC.1399.793).

## 3 Measurements of oxidative stress biomarkers in brain homogenate

### 3.1 Evaluation of brain lipid peroxidation (LPO)

As described in Yagi’s method, thiobarbituric acid (TBA) reacts against lipid peroxide agents; hence it is beneficial to measure LPO. Homogenates were mixed with 20% trichloro acetic acid, and the consequence precipitate was dissolved in sulphuric acid. Next 0.2% TBA in 2 M sodium sulphate was added, and the mixture was heated in bain-marie for 30 min. LPO was extracted using 1-butanol, and the optical density was measured at 532 nm [21].

### 3.2 Evaluation of brain total antioxidant capacity (TAC)

The TAC was measured using the ferric reducing ability of plasma method. This method is based on the capacity of a sample to transform Fe^3+^ to Fe^2+^ in the presence of Tripyridyl-*s*-triazine (TPTZ). The interaction between Fe^2+^ and TPTZ results in a blue complex. The optical density was measured at 593 nm according to a calibration curve attained by serial concentrations of FeSO_4_ [22].

### 3.3 Evaluation of brain total thiol groups (TTG)

By a spectrophotometric technique presented by Hu and Dillard [23] and using DTNB (Ellman’s reagent), brain homogenates TTG were measured: 1 mL of Tris buffer (250 mM and EDTA 2 mM) was added to 50 mL of homogenate. Following the addition of 20 mL of DTNB, a yellow complex was evaluated at 412 nm.

### 3.4 Evaluation of brain catalase (CAT) activity

According to the manufacturer’s instructions, CAT activity was measured using the Kiazist kit, Iran (Kiazist, KCAT-96). The protocol is based on the degradation of hydrogen peroxide (H_2_O_2_) in the presence of methanol and the formation of formaldehyde which reacts with purpald (maximum absorbance at 540 nm). Briefly, 20 mL of homogenates were mixed with 100 mL of CAT assay buffer and 30 mL of methanol in a 96-well plate. Next 20 mL of substrate were added, and the plate was covered for 20 min and put at ambient temperature. Later, the reaction was stopped with the stop solution, and 30 mL of chromogen were added. After 10 min of being kept in darkness, optical density was measured. The activity was calculated based on the following equation reported as nmol/min/mg of protein.

{\rm{CAT\; activity}}=\frac{{\rm{\mu }}{\rm{M\; of\; purpald}}}{20}\times \frac{0.24}{0.02}\times {\rm{D}}{\rm{ilution\; coefficient}}.]

### 3.5 Superoxide dismutase (SOD) activity

We determined the SOD activity using a commercial kit (SOD activity pianist; Iran) based on the manufacturer’s instructions. This method relies on Mn-SOD’s ability to inhibit resazurin conversion to resorufin when involved with reducing superoxide radicals produced by xanthine/xanthine oxidase.

### 3.6 Evaluation of brain total protein content

Total protein concentration was measured by Bradford reagent. One hundred milligrams of Coomassie Brilliant Blue G-250 were dissolved in 50 mL of 95% ethanol and added to 100 mL of 85% phosphoric acid as Bradford reagent. Protein contents of the homogenates were measured at 595 nm against a bovine serum albumin [24].

### 3.7 Evaluation of histopathological changes in brain

Brain samples submerged in 10% neutral buffered formalin solution were dehydrated in graded ethanol concentrations, immersed in xylene, and embedded in paraffin. Sections were cut at 10 mm on a microtome and fixed and stained using cresyl violet. Afterwards, the hippocampus sections recorded with a camera (Nikon E800, Japan) linked to a microscope. The pathological changes were examined for each animal by scanning five serial coronal sections at 40× magnifications, and bright cells in the CA1 region were counted via ImageJ programming. An experienced histologist who was blind to the study groups and treatments carried out the microscopic assessments.

### 3.8 Statistical analysis

Assays were performed in triplicate. The outcomes were communicated as the mean value ± standard error of the mean, and the Kolmogorov–Smirnov test confirmed normal distribution on all measures (*P* ≥ 0.05). The groups were compared using a one-way ANOVA test. The statistical investigation and data visualisation was carried out utilising “GraphPad Prism version 8.0.0 for Windows, GraphPad Software, San Diego, California USA, www.graphpad.com.” We used violin plots to better demonstrate how data are distributed due to erroneous and non-homogeneous nature of biochemical experiments. A *P*-value less than 0.05 was taken as the significance at a minimum level for all tests.

## 4 Results

### 4.1 Effects of crocin and crocin loaded niosomes (NC) on brain LPO

**Figure 1:**
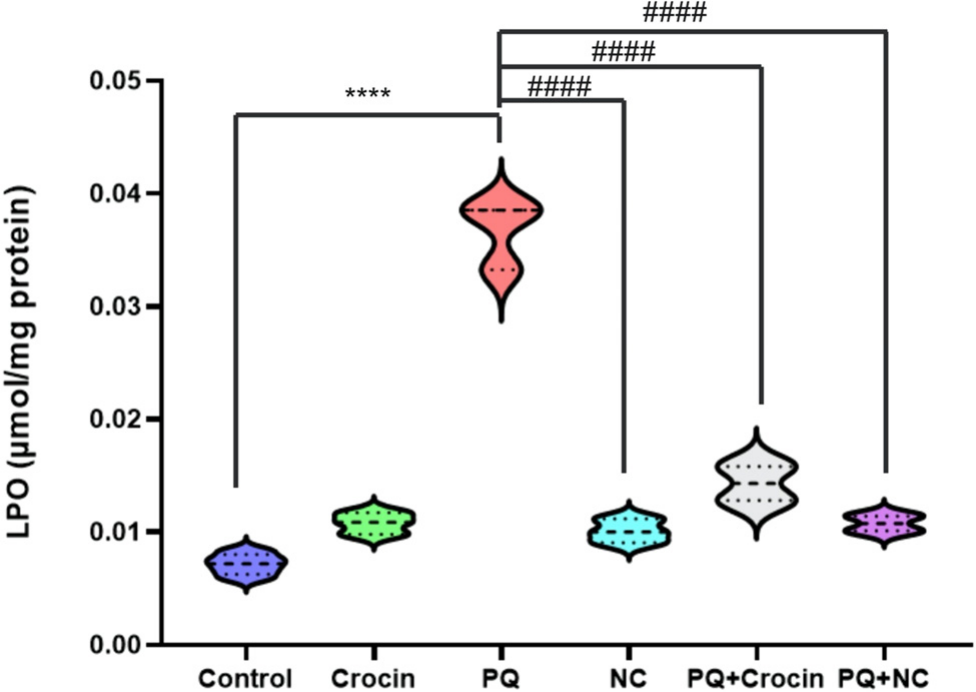
Effects of crocin and NC on LPO in rat brain tissue. PQ: Paraquat (5 mg/kg/day for 7 days by IP); Crocin (20 mg/kg/day for 7 day by IP); NC: crocin loaded niosomes (20 mg/kg/day for 7 day by IP). **** indicates significant difference with control (*P* < 0.0001), and ^####^ indicates significant difference with group P (*P* < 0.0001).

The LPO level revealed that PQ induced a significant increase in LPO in the brain compared to control (*P* < 0.0001). Treatment with crocin and NC dropped LPO levels promisingly (*P* < 0.0001); however, there was a mild difference between groups crocin and NC (Figure 1).

### 4.2 Effects of crocin and NC on brain TAC

**Figure 2:**
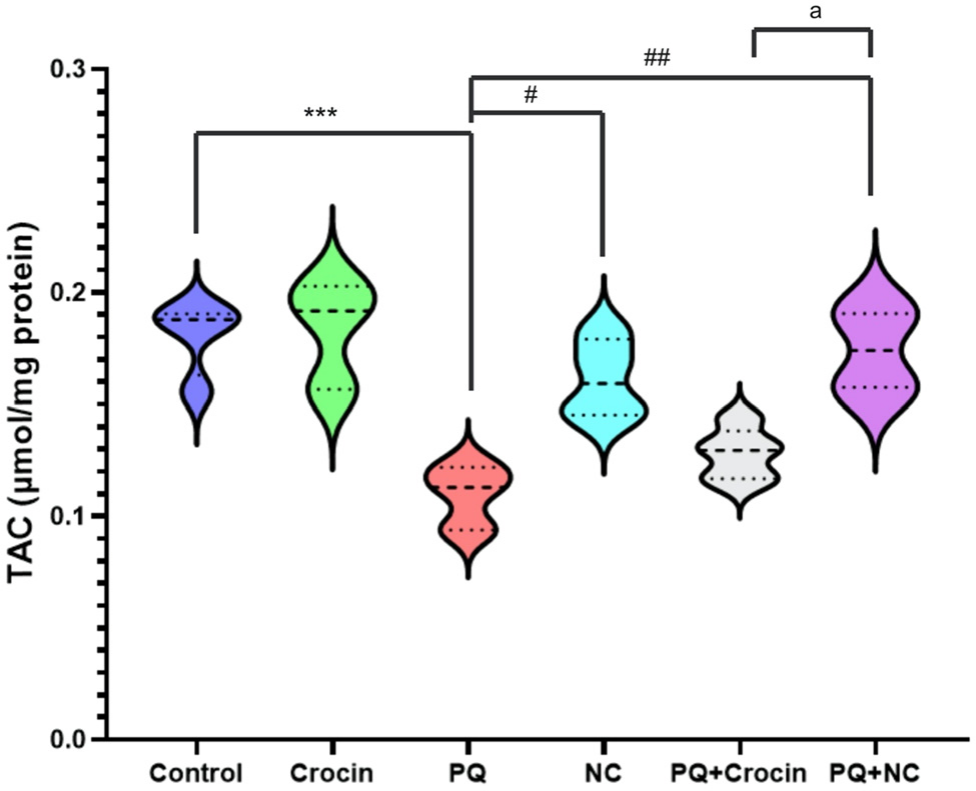
Effects of crocin and NC on TAC of brain tissue. PQ: Paraquat (5 mg/kg/day for 7 days by IP); Crocin (20 mg/kg/day for 7 days by IP); NC: crocin loaded niosomes (20 mg/kg/day for 7 day by IP). **** indicates significant difference with control (*P* < 0.0001), and ^##^ (*P* < 0.01), ^###^ (*P* < 0.001) indicates significant difference with group P (*P* < 0.001). aa indicates significant difference with Crocin and crocin loaded niosomes treatment group (*P* < 0.01).

TAC assessment revealed a significant difference between group PQ and control (*P* < 0.001). Treatments with crocin or NC resulted in minor differences in TAC levels compared with control, which was not significant. Also, NC (*P* < 0.01) attenuated the effects of PQ on TAC levels in brain tissue, and apparently, the nanoformulation of crocin was more promising. A significant difference (*P* < 0.05) was seen in the effects of crocin and NC on TAC levels (Figure 2).

### 4.3 Effects of crocin and NC on TTG

**Figure 3:**
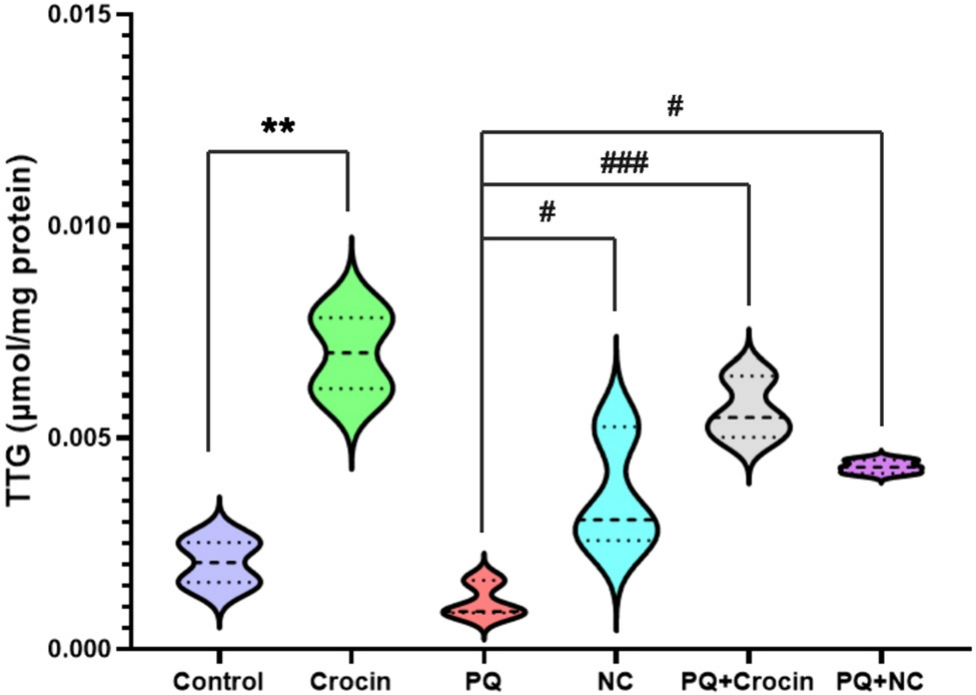
Effects of crocin and NC on TTG of brain tissue. PQ: Paraquat (5 mg/kg/day for 7 days by IP); Crocin (20 mg/kg/day for 7 days by IP); NC: crocin loaded niosomes (20 mg/kg/day for 7 days by IP). ** indicates significant difference with control (*P* < 0.01), and ^#^ (*P* < 0.05), ^###^ (*P* < 0.001) indicates significant difference with group PQ (*P* < 0.001).

The evaluations indicated that crocin had a significant effect on TTG compared to control (*P* < 0.01). The levels of TTG in PQ-challenged rats decreased significantly (compared to Crocin, NC, and the control group), which was compensated by Crocin (*P* < 0.001) and NC (*P* < 0.05). Both crocin and NC had restored the TTG levels to baseline in rats challenged with PQ (Figure 3). Also, there was a mild difference between TTG in PQ + Crocin and PQ + NC.

### 4.4 Effects of crocin and NC on brain CAT activity

**Figure 4:**
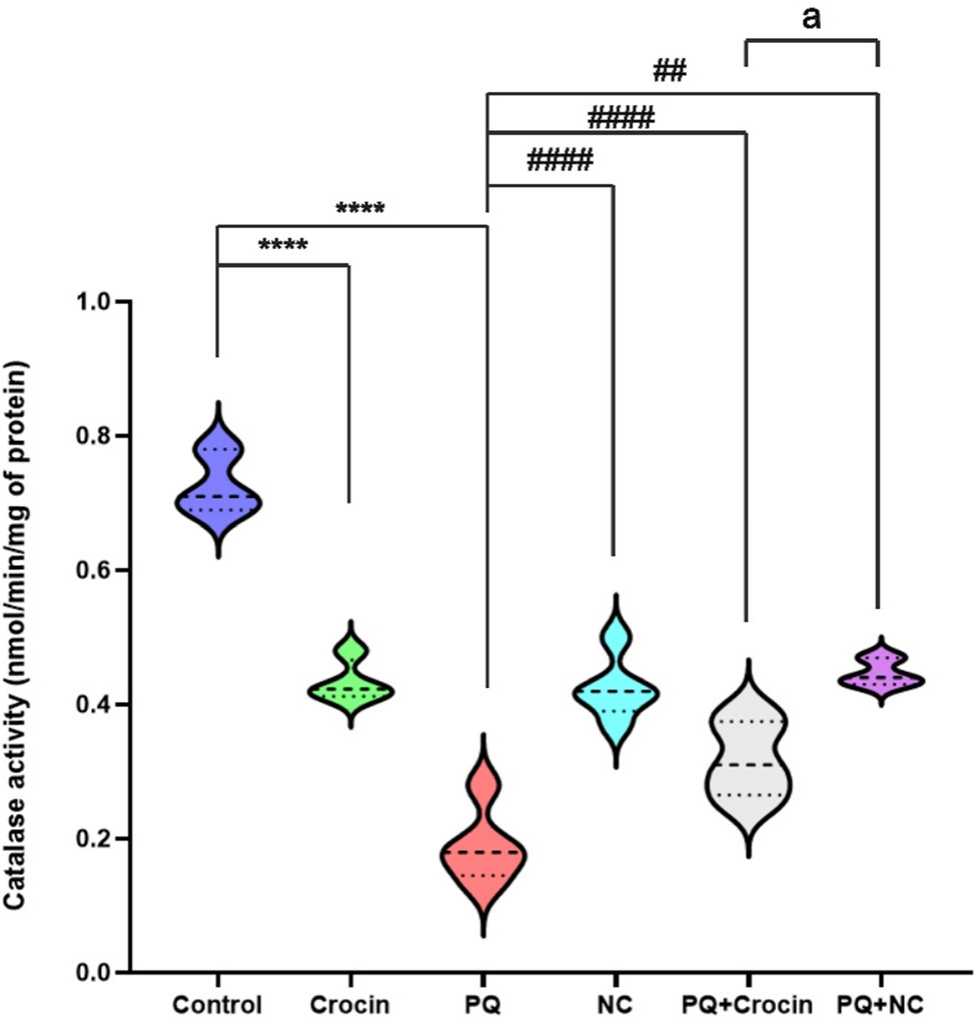
Effects of crocin and NC on CAT activity of the brain. PQ: Paraquat (5 mg/kg/day for 7 days by IP); Crocin (20 mg/kg/day for 7 days by IP); NC: crocin loaded niosomes (20 mg/kg/day for 7 days by IP). **** indicates significant difference with control (*P* < 0.0001), and ^##^ (*P* < 0.01), ^####^ (*P* < 0.0001) indicates significant difference with group PQ (*P* < 0.001). a indicates significant difference with crocin and crocin loaded niosomes treatment group (*P* < 0.05).

The assessment of CAT activity revealed a significant difference between the control and all other groups. The group treated with crocin (*P* < 0.0001) and NC (*P* < 0.0001) displayed substantial improvement in CAT activity compared to PQ. The activity of CAT in PQ + Crocin (*P* < 0.0001) and PQ + NC (*P* < 0.01) groups showed a significant increase compared to PQ (Figure 4). The critical difference (*P* < 0.05) was seen in the effects of crocin and NC on CAT levels.

### 4.5 Effects of crocin and NC on brain SOD *activity*

**Figure 5:**
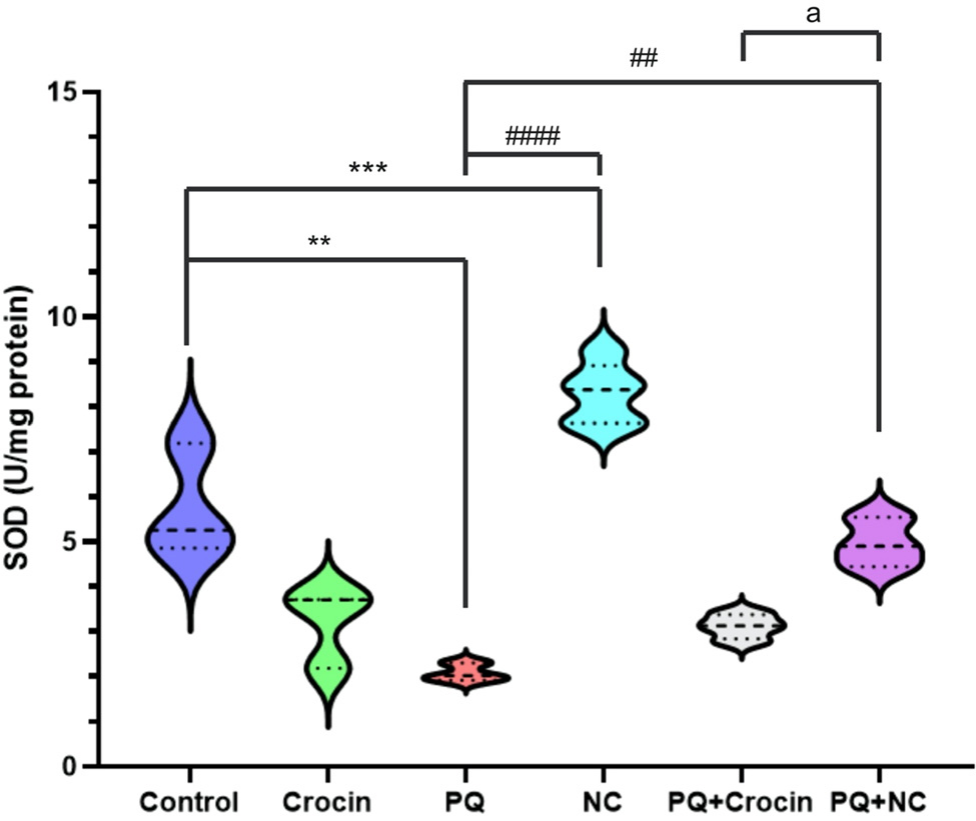
Effects of crocin and NC on SOD activity of the brain. PQ: Paraquat (5 mg/kg/day for 7 days by IP); Crocin (20 mg/kg/day for 7 days by IP); NC: crocin loaded niosomes (20 mg/kg/day for 7 days by IP). *** (*P* < 0.001), **** (*P* < 0.0001) indicates significant difference with control), and ^##^ (*P* < 0.01) indicates significant difference with group PQ.

The assessment of SOD activity revealed a significant difference between the control and PQ (*P* < 0.01) and NC (*P* < 0.001) groups. The group treated with NC displayed significantly improved CAT activity compared to PQ (*P* < 0.05). The activity of CAT in PQ + NC groups showed a significant increase compared to PQ (*P* < 0.01) (Figure 5). A significate difference (*P* < 0.05) was established between PQ + Crocin and PQ + NC in CAT activity.

### 4.6 Effects of crocin and NC on PQ-induced histological changes in the brain

**Figure 6:**
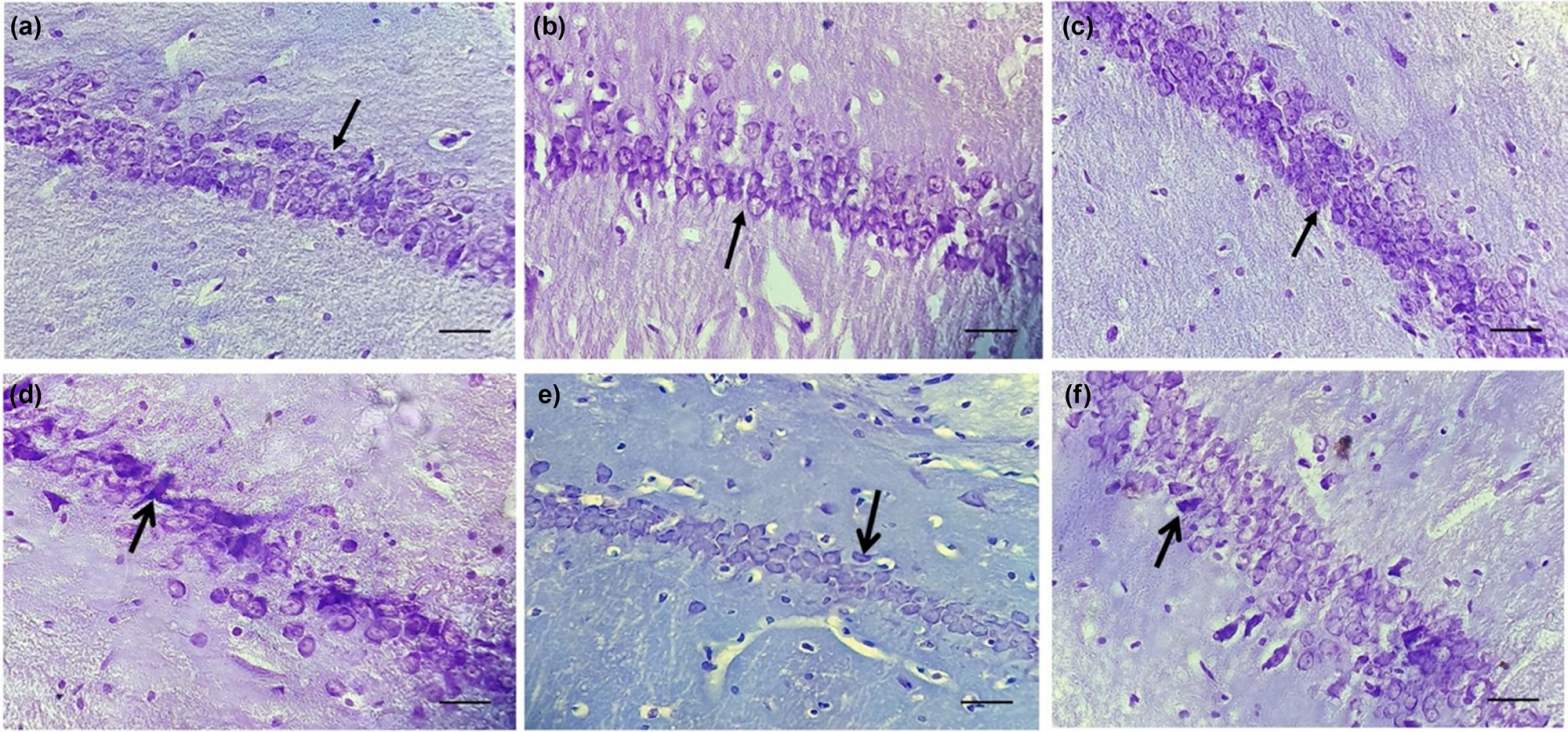
Effects of crocin and NC on histopathological changes in the CA1 region of the hippocampus. Assessed by cresyl violet staining, 40× magnification. Rats were exposed to paraquat + crocin or NC for 7 days. (a) Control, (b) Crocin, (c) NC (d) Paraquat, (e) Paraquat + Crocin, and (f) Paraquat + NC. Arrows indicate cell density in the hippocampal CA1 region.

**Figure 7:**
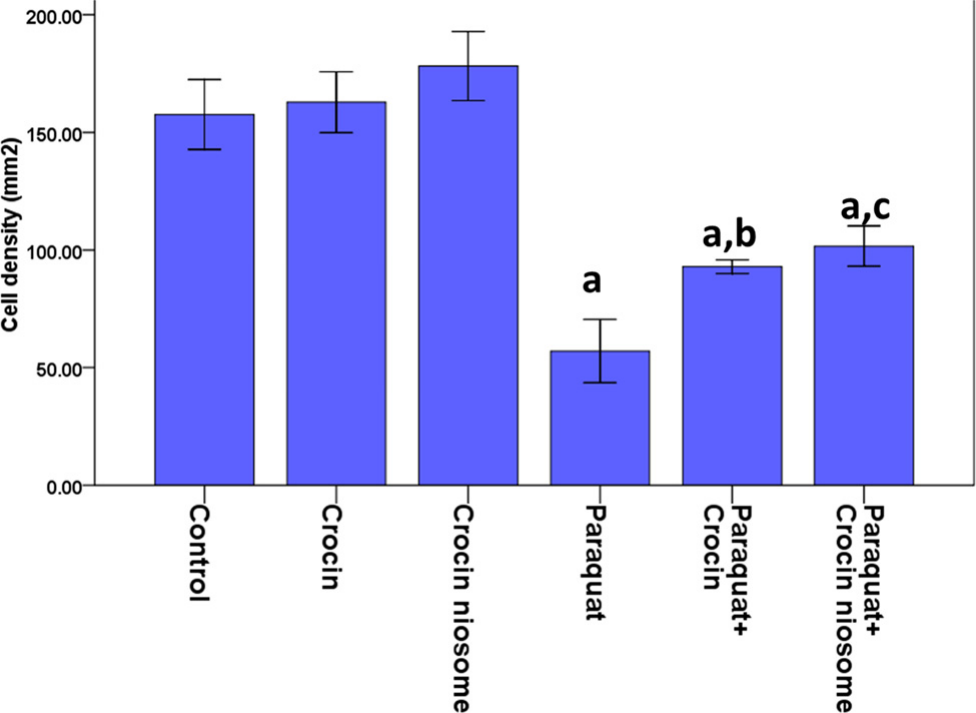
Effects of crocin and NC on histopathological changes in the CA1 region of the hippocampus. Results are expressed as Mean value ± SEM. a indicates a significant difference with group Control (*P* < 0.001); and b (*P* < 0.01) as well c (*P* < 0.001) indicate a significant difference with group Paraquat.

Histological evaluations revealed that the PQ-induced (Figures 6d and 7) rats displayed a significant decrease in cell density in the CA1 region of the hippocampus compared to normal (Figures 6a and 7), crocin (Figures 6b and 7), and NC (Figures 6c and 7) (*P* < 0.001) groups. Treatments with crocin (Figures 6e and 7) (*P* < 0.01) and NC (Figures 6f and 7) (*P* < 0.001) plus PQ led to an enhancement in cell density of CA1 compared to the PQ group. Although treatment with crocin or NC showed no difference in cell count, the bulk and nanoformulations of crocin presented different effect levels in PQ-toxicity rats (Figures 6 and 7). No difference was seen between the crocin and NC groups compared to the control group (Figures 6 and 7).

## 5 Discussion

In the present study, we investigated the antioxidant efficacy of crocin and NC in the brain of rats treated with PQ. It would also be helpful to apply higher doses to compare the results with a broader range of studies. Antioxidant, anti-apoptotic, and anti-inflammatory properties are the primary mechanisms linking Crocin to its neuroprotective effects. There is some evidence that Crocin is a promising treatment option for neurodegenerative diseases, as it appears to cause fewer adverse effects than existing medications. Crocin is a poor pharmacotherapeutic agent because of its low dissolution rate, low bioavailability, and rapid elimination [25]. This crocin deficiency may be addressed by nano-crocin or nanocarriers like NC with higher bioavailability as our study confirmed this issue by showing some biochemical and histopathological evidence to support it. The biological effects of crocin against toxicants in various studies have been attributed to their antioxidant and anti-inflammatory properties [14,15,17]. Our results show that exposure to PQ for 1 week led to brain damage through an imbalance in redox status and induction of oxidative stress, as demonstrated by elevated LPO, decreased TAC and TTG content, and CAT and SOD activity. These findings are supported by cresyl violet staining of the hippocampus, showing a reduction in cells present in the CA1 region. Cyclic single-electron reduction/oxidation is an essential part of events accounting for the possible mechanism of PQ toxicity [26]. We also found the improvement in LPO, TAC, and TTG using crocin and NC suggesting that crocin treatment could protect the brain against the unfavourable effects of PQ by removing free radicals and improving the oxidative status in the brain. Also, NC improved TAC, CAT, and SOD activity levels, indicating noticeable differences with bulk forms of crocin. Surprisingly, crocin or NC administration alone did not significantly differentiate the effects in our work compared to the control. It can be said that the initial oxidative stress may not differ from the antioxidant capacity of crocin in the applied doses. However, when oxidative stress develops significantly – such as PQ toxicity – the antioxidant effect increases or is overcome during treatment, and finally, crocin and NC can act as neuroprotective antioxidants. These results are consistent with our results from other field studies on the crocin [15,27,28]. The applied NC showed 140 nm in diameter and -23 mV and a burst release during the first 8 h. Based on the literature, NC having diameters smaller than 200 nm can proceed through the blood–brain barrier (BBB) [29]. Hence, the NC have successfully reached the neural tissue in total or partially. On the other hand, the passage of nanoparticles to the brain involves an electrostatic interaction between a positively-charged agent and the negatively-charged cell membrane at the BBB, which is introduced as adsorptive mediated endocytosis. The positive zeta potential of particles helps promote the niosome passage through BBB [30]. The negative charge of the applied NC might not allow the BBB passage but facilitate the retaining time in circulation. This might explain the similarity of the results achieved by the PQ + Crocin and PQ + NC groups. It has been reported that crocin can be safely administered orally for prolonged periods of time to rats at high doses (50–100 mg/kg) [31]. Even though the amounts we used in the current study are much lower (20 mg/kg), there were some unfavourable results following IV injection of crocin, and we observed increased LPO. But some studies have examined the antioxidant effects of crocin injections intraperitoneally against oxidative damage caused by 6-hydroxydopamine [32]. LPO is a critical mechanism in the damaging process of PQ in multiple organs [5,33]. This mechanism may also be applied to neural damage. Mitochondria are a site involved in environmental pollutants [34]. Due to energy requirements, significant consumption of oxygen, and mitochondrial abundance, the brain and neurons are susceptible to oxidative stress. Additionally, neurons are rich in polyunsaturated fatty acids, which are especially susceptible to induced LPO [3]. The substantial evidence indicating that Crocin possesses neuro-pharmacological viability by different exploration standards leads to the conclusion that Crocin possesses direct antioxidant, antiapoptotic, anti-inflammatory, and antiproliferative properties [35]. On the other hand, Crocin was found to raise the level of dopamine in the brain during experiments with Parkinson’s disease [36]. Considering the link between PQ and Parkinson’s, Crocin and NC neutralise the oxidative and destructive effects of PQ, making them potentially beneficial for Parkinson’s treatment [37]. Also, numerous studies have confirmed that Crocin works similar to fluoxetine and imipramine in treating depressive disorders [38]. All attempts to improve the bioavailability and pharmacotherapeutic properties of crocin using nanotechnology will assist in reducing the management and treatment costs for both neurodegenerative and psychiatric patients by providing cheap, available, and effective treatment.

## 6 Limitations and future perspective

There are some limitations in the experimental design of the present study, such as various formulations of crocin, especially with different physicochemical characteristics (such as zeta potential, size, and loading percent). In this study, crocin and NC were found to have neuroprotective properties and the ability to modulate oxidative stress conditions in the brain. In many neurodegenerative disorders, including Alzheimer’s, Parkinson’s, and strokes, oxidative stress plays an essential role in their pathogenesis. According to the findings of this study, crocin and especially NC may help treat these neurodegenerative diseases by modulating the effects of oxidative stress in the brain.

## 7 Conclusion

Our findings demonstrate that NC is a promising agent for preventing the brain damage resulting from PQ – induced neurotoxicity. Crocin and NC significantly modulate oxidative damage in the brain.

## References

[1] Amirshahrokhi K, Bohlooli S. Effect of methylsulfonylmethane on paraquat-induced acute lung and liver injury in mice. Inflammation. 2013;36(5):1111–21. 10.1007/s10753-013-9645-8 23595869

[2] Nunes ME, Müller TE, Braga MM, Fontana BD, Quadros VA, Marins A, et al. Chronic treatment with paraquat induces brain injury, changes in antioxidant defenses system, and modulates behavioral functions in zebrafish. Mol Neurobiol. 2017;54(6):3925–34. 10.1007/s12035-016-9919-x 27229491

[3] McCormack AL, Atienza JG, Johnston LC, Andersen JK, Vu S, Di Monte DA. Role of oxidative stress in paraquat‐induced dopaminergic cell degeneration. J Neurochem. 2005;93(4):1030–7. 10.1111/j.1471-4159.2005.03088.x 15857406

[4] Bus JS, Gibson JE. Paraquat: model for oxidant-initiated toxicity. Environ Health Perspect. 1984;55:37–46. 10.1289/ehp.845537 PMC1568364 6329674

[5] Bateman D. Pharmacological treatments of paraquat poisoning. Hum Toxicol. 1987;6(1):57–62. 10.1177/096032718700600109 3546087

[6] Berry C, La Vecchia C, Nicotera P. Paraquat and Parkinson’s disease. Cell Death Differ. 2010;17(7):1115–25. 10.1038/cdd.2009.217 20094060

[7] Kin K, Yasuhara T, Kameda M. Animal models for Parkinson’s disease research: trends in the 2000s. Int J Mol Sci. 2019;20(21):5402. 10.3390/ijms20215402 PMC6862023 31671557

[8] José Bagur M, Alonso Salinas GL, Jiménez-Monreal AM, Chaouqi S, Llorens S, Martínez-Tomé M, et al. Saffron: An old medicinal plant and a potential novel functional food. Molecules. 2018;23(1):30. 10.3390/molecules23010030 PMC5943931 29295497

[9] Gregory MJ, Menary RC, Davies NW. Effect of drying temperature and air flow on the production and retention of secondary metabolites in saffron. J Agric Food Chem. 2005;53(15):5969–75. 10.1021/jf047989j 16028982

[10] Lambrianidou A, Koutsougianni F, Papapostolou I, Dimas K. Recent advances on the anticancer properties of Saffron (Crocus sativus L.) and its major constituents. Molecules. 2021;26(1):86. 10.3390/molecules26010086 PMC7794691 33375488

[11] Yang R, Yang L, Shen X, Cheng W, Zhao B, Ali KH, et al. Suppression of NF-κB pathway by crocetin contributes to attenuation of lipopolysaccharide-induced acute lung injury in mice. Eur J Pharmacol. 2012;674(2–3):391–6. 10.1016/j.ejphar.2011.08.029 21925167

[12] Lin J-K, Wang C-J. Protection of crocin dyes on the acute hepatic damage induced by aflatoxin B1 and dimethylnitrosamine in rats. Carcinogenesis. 1986;7(4):595–9. 10.1093/carcin/7.4.595 2870820

[13] Jnaneshwari S, Hemshekhar M, Santhosh MS, Sunitha K, Thushara R, Thirunavukkarasu C, et al. Crocin, a dietary colorant mitigates cyclophosphamide-induced organ toxicity by modulating antioxidant status and inflammatory cytokines. J Pharm Pharmacol. 2013;65(4):604–14. 10.1111/jphp.12016 23488790

[14] El-Beshbishy HA, Hassan MH, Aly HA, Doghish AS, Alghaithy AA. Crocin “saffron” protects against beryllium chloride toxicity in rats through diminution of oxidative stress and enhancing gene expression of antioxidant enzymes. Ecotoxicol Environ Saf. 2012;83:47–54. 10.1016/j.ecoenv.2012.06.003 22766413

[15] Altinoz E, Erdemli M, Gul M, Aksungur Z, Gul S, Bag H, et al. Neuroprotection against CCl4 induced brain damage with crocin in Wistar rats. Biotechnic Histochem. 2018;93(8):623–31. 10.1080/10520295.2018.1519725 30273072

[16] Altinzo E, Oner Z, Elbe H, Vardi N. Neuro-protective effects of crocin on brain and cerebellum tissues in diabetic rats. Afr J Trad Complemen Altern Med. 2014;11(6):33–9.

[17] Fathimoghadam H, Farbod Y, Ghadiri A, Fatemi R. Moderating effects of crocin on some stress oxidative markers in rat brain following demyelination with ethidium bromide. Heliyon. 2019;5(2):e01213. 10.1016/j.heliyon.2019.e01213 PMC6378371 30815598

[18] Du P, Qian Z-y. Studies on the absorption and excretion of crocin-1 in rats. Chin N Drugs J. 2004;13:802–4.

[19] Mirhadi E, Nassirli H, Malaekeh-Nikouei B. An updated review on therapeutic effects of nanoparticle-based formulations of saffron components (safranal, crocin, and crocetin). J Pharm Invest. 2020;50(1):47–58.

[20] Samarghandian S, Azimi-Nezhad M, Borji A, Farkhondeh T. Effect of crocin on aged rat kidney through inhibition of oxidative stress and proinflammatory state. Phytother Res. 2016;30(8):1345–53. 10.1002/ptr.5638 27279282

[21] Yagi K. Simple assay for the level of total lipid peroxides in serum or plasma. Methods Mol Biol. 1998;108:101–6. 10.1385/0-89603-472-0:101 9921519

[22] Benzie IF, Strain J. Ferric reducing/antioxidant power assay: direct measure of total antioxidant activity of biological fluids and modified version for simultaneous measurement of total antioxidant power and ascorbic acid concentration. Methods Enzymol. 1999;299:15–27. 10.1016/s0076-6879(99)99005-5 9916193

[23] Hu M, Dillard C. Plasma SH and GSH measurement. Methods in Enzymology. 1994;233:385–7.

[24] Jones CG, Hare JD, Compton SJ. Measuring plant protein with the Bradford assay. J Chem Ecol. 1989;15(3):979–92. 10.1007/BF01015193 24271900

[25] Ahmed S, Hasan MM, Heydari M, Rauf A, Bawazeer S, Abu-Izneid T, et al. Therapeutic potentials of crocin in medication of neurological disorders. Food Chem Toxicol. 2020;145:111739. 10.1016/j.fct.2020.111739 32916219

[26] Morán JM, Ortiz-Ortiz MA, Ruiz-Mesa LM, Fuentes JM. Nitric oxide in paraquat-mediated toxicity: A review. J Biochem Mol Toxicol. 2010;24(6):402–9. 10.1002/jbt.20348 21182169

[27] Altinoz E, Ozmen T, Oner Z, Elbe H, Erdemli M, Bag H. Saffron (its active constituent, crocin) supplementation attenuates lipid peroxidation and protects against tissue injury. Bratisl Lek Listy. 2016;117(7):381–7. 10.4149/bll_2016_075 27546539

[28] Erdemli Z, Erdemli ME, Gul M, Altinoz E, Gul S, Kocaman G, et al. Ameliorative effects of crocin on the inflammation and oxidative stress-induced kidney damages by experimental periodontitis in rat. Iran J Basic Med Sci. 2021;24(6):825. 10.22038/ijbms.2021.55875.12499 PMC8487597 34630960

[29] Shilo M, Sharon A, Baranes K, Motiei M, Lellouche J-PM, Popovtzer R. The effect of nanoparticle size on the probability to cross the blood-brain barrier: an in-vitro endothelial cell model. J Nanobiotechnol. 2015;13(1):1–7. 10.1186/s12951-015-0075-7 PMC4359781 25880565

[30] Honary S, Zahir F. Effect of zeta potential on the properties of nano-drug delivery systems-a review (Part 1). Tropical J Pharm Res. 2013;12(2):255–64.

[31] Margaritis I, Angelopoulou K, Lavrentiadou S, Mavrovouniotis IC, Tsantarliotou M, Taitzoglou I, et al. Effect of crocin on antioxidant gene expression, fibrinolytic parameters, redox status and blood biochemistry in nicotinamide-streptozotocin-induced diabetic rats. J Biol Res-Thessaloniki. 2020;27(1):1–15. 10.1186/s40709-020-00114-5 PMC7053078 32161725

[32] Hosseini M, Rajaei Z, Alaei H, Tajadini M. The effects of crocin on 6-OHDA-induced oxidative/nitrosative damage and motor behaviour in hemiparkinsonian rats. Malaysian J Med Sci MJMS. 2016;23(6):35. 10.21315/mjms2016.23.6.4 PMC5181990 28090177

[33] Jouzdani AF, Ganjirad Z, Firozian F, Soliemani-Asl S, Ranjbar A. Protective effects of N-acetylcysteine niosome nanoparticles on paraquat-induced nephrotoxicity in male rats. Pharm Nanotechnol. 2022;10(2):137–45. 10.2174/2211738510666220214102034 35156589

[34] Yousefsani BS, Pourahmad J, Hosseinzadeh H. The mechanism of protective effect of crocin against liver mitochondrial toxicity caused by arsenic III. Toxicol Mech Methods. 2018;28(2):105–14. 10.1080/15376516.2017.1368054 28812436

[35] Kermanshahi S, Ghanavati G, Abbasi-Mesrabadi M, Gholami M, Ulloa L, Motaghinejad M, et al. Novel neuroprotective potential of crocin in neurodegenerative disorders: an illustrated mechanistic review. Neurochem Res. 2020;45(11):2573–85. 10.1007/s11064-020-03134-8 32940861

[36] Iranshahy M, Javadi B, Sahebkar A. Protective effects of functional foods against Parkinson’s disease: A narrative review on pharmacology, phytochemistry, and molecular mechanisms. Phytother Res. 2022;36(5):1952–89. 10.1002/ptr.7425 35244296

[37] Mou L, Ding W, Fernandez-Funez P. Open questions on the nature of Parkinson’s disease: from triggers to spreading pathology. J Med Genet. 2020;57(2):73–81. 10.1136/jmedgenet-2019-106210 31484719

[38] Siddiqui SA, Ali Redha A, Snoeck ER, Singh S, Simal-Gandara J, Ibrahim SA, et al. Anti-depressant properties of crocin molecules in Saffron. Molecules. 2022;27(7):2076. 10.3390/molecules27072076 PMC9000812 35408474

